# Who and Where: A Socio-Spatial Integrated Approach for Community-Based Health Research

**DOI:** 10.3390/ijerph15071375

**Published:** 2018-06-30

**Authors:** Jeanne-Marie R. Stacciarini, Raffaele Vacca, Liang Mao

**Affiliations:** 1 College of Nursing, University of Florida, Gainesville, FL 32610, USA; jeannems@ufl.edu; 2 Department of Sociology and Criminology & Law, University of Florida, Gainesville, FL 32611, USA; r.vacca@ufl.edu; 3 Department of Geography, University of Florida, Gainesville, FL 32611, USA

**Keywords:** community health, rural Latino immigrants, mental well-being, network analysis, spatial statistics, intervention

## Abstract

Social and spatial characteristics of a population often interact to influence health outcomes, suggesting a need to jointly analyze both to offer useful insights in community health. However, researchers have used either social or spatial analyses to examine community-based health issues and inform intervention programs. We propose a combined socio-spatial analytic approach to develop a *social network with spatial weights* and a *spatial statistic with social weights*, and apply them to an ongoing study of mental and physical well-being of rural Latino immigrants in North Florida, USA. We demonstrate how this approach can be used to calculate measures, such as social network centrality, support contact dyads, and spatial kernel density based on a health survey data. Findings reveal that the integrated approach accurately reflected interactions between social and spatial elements, and identified community members (*who*) and locations (*where*) that should be prioritized for community-based health interventions.

## 1. Introduction

In the last ten years, community-based health researchers have increasingly used either social or spatial analytics to examine health issues and inform intervention programs [1,2,3,4,5]. While this approach has enabled researchers to reveal social or geographic connections within a population, intervention programs based on this approach have encountered two primary problems: targeted individuals can be misidentified due to incomplete social network information, and a targeted intervention site may be visited by very few people from the population of interest. Problems of this type can largely reduce the effectiveness of community-based health interventions. In real life, social and spatial dimensions in a community tend to interact and should not be treated as mutually exclusive in analysis [6]. For instance, social connections may change with people’s spatial locations, and in turn people’s spatial activities are shaped by their social connections [7,8]. As a result of the interaction between these factors, in the past twenty years, social scientists have increasingly called for more integration between social and spatial information [9], including in public health studies and interventions [10]. However, to date, little attention has been paid to integrating social and spatial analytics to understand community health and inform “who” and “where” an intervention should target.

Thus, to reflect ongoing interactions between social and spatial elements in the same community, this methodological article aims to present a unique socio-spatial approach that aims to study data using: (1) a *social network with spatial weights*; and (2) a *spatial statistic with social weights*. The data under study were derived from an exploratory research conducted among Latino immigrants in rural North Florida, an area of interest for interventions to promote mental and physical well-being. Given that rural populations tend to be less mobile than their urban living counterparts and have limited places to visit in their communities, the proposed integrative approach could well fit these hard-to-reach populations and better inform health intervention design in rural communities.

### 1.1. A Review of Social and Spatial Analyses in Community-Based Health Research

#### 1.1.1. Social Analysis in Community-Based Health Research

Because social relationships can dramatically impact the health outcomes of a community [3,11], social analytics have been used frequently in community-based health research, such as sentiment analysis, text analysis, and social network analysis [12,13,14]. One type of social analytics—social network analysis—uses a finite number of *nodes* to represent individuals and their characteristics such as age, gender, and occupation. *Links* are assigned between nodes to represent either social relationships or interactions, and *link weights* are used to measure characteristics of these connections, such as the strength of social relationships or the frequency of interaction. Researchers collect data about participants from community-wide social/health surveys that obtain information about social contacts, such as characteristics of respondents’ friends, sources of professional help, and frequency of interaction with other. Using these data, researchers describe the network structure by calculating variables such as the number of links attached to each node (a measure known as degree centrality) or identifying cohesive subgroups of individuals who are strongly connected to each other. Next, statistical models, such as logistic regression and multilevel modeling, are used to explore associations between network measures and health outcomes. Findings may also be used to rank individuals and groups according to health status or network characteristics, and identify those who can benefit most from, or can be most helpful for, interventions [15]. Most social network studies of community health are aspatial: they locate people in a topological space of social relationships, where spatial arrangements of individuals are not considered.

#### 1.1.2. Spatial Analysis in Community-Based Health Research

Spatial analysis aims to highlight a relationship among participants’ residential location, movement between locations, and health outcomes, such as depression, obesity, and access to healthcare [2,4,5]. Data collection may include searching hospital records to discover how participants access health care, asking participants to complete a diary of their travels or a survey, or examining their location data from social networking websites or GPS loggers. Next, a range of spatial statistics are used to summarize participants’ spatial activities [16], such as a two dimensional ellipse that envelopes all locations visited by each participant, and a spatial density map that shows clusters of locations visited by all participants [17]. Findings often reveal areas associated with negative health outcomes, and help researchers identify locations that may benefit from public health interventions. In most of these spatial studies of community health, the spatial relationships between individuals are emphasized (for instance their geographic proximity), but the social attributes or connections between them are neglected.

Although a large body of research exists that uses either social or spatial analysis to examine health outcomes, the findings are limited because social and spatial dimensions have ongoing, dynamic relationships that are better examined in tandem [18]. The following section elaborates the proposed socio-spatial integrated approach: (1) how to integrate spatial weights into social network analysis; and (2) how to assimilate social weights into spatial statistics.

## 2. Methods 

### 2.1. Integrating Spatial Weights with Social Network

Based on evidence that suggests a strong correlation between geographic proximity and social ties [8,19], we constructed a spatially-weighted social network that uses nodes to represent individuals as well as their demographic-, social-, and health-related characteristics. The links between nodes represent social connections inferred from spatial relationships between individuals. For example, if significant overlap exists in the locations visited by two individuals (also known as *co-location*), then we can infer that those individuals are likely to be socially linked. The weights on links indicate the degree of co-location between nodes (or the likelihood of social connection), and are estimated by following steps. Each individual *i* is assigned a 1 × *L* vector ***V_i_***, where *L* is the total number of locations in a study region. Each element in ***V_i_*** records the probability of individual *i* visiting a location, and hereinafter the vector ***V_i_*** is referred to as the *location vector* for *i*. Using the location vectors of two individuals *i* and *j*, the degree of co-location *w_ij_* between them is measured as the cosine of angle between ***V_i_*** and ***V_j_*** in a *L*-dimensional location space, commonly known as cosine similarity:

(1) $$w_{ij}=cos\theta_{ij}=\frac{V_{i}\times V_{j}}{∥V_{i}∥∥V_{j}∥}=\frac{\sum_{k=1}^{L}V_{ik}V_{kj}}{\sqrt{\sum_{k}V_{ik}^{2}}\sqrt{\sum_{k}V_{jk}^{2}}}\;\;\in \left[0,1\right]$$

If the two location vectors are similar, the angle between them is close to zero and the cosine value is near to 1, indicating a high degree of co-location and a great probability of social connection. 

Once a spatially-weighted social network is built, network measures can be derived to identify key individuals or groups for intervention purposes. For example, a key lay-community leader in an intervention should be highly connected to other members in the network. The individual connectivity can be measured as a weighted degree centrality of each node, the sum of all weights on the links associated with the node:

(2) $$DC_{i}=\overset{n}{\underset{j=1}{\sum }}w_{ij}=\overset{n}{\underset{j=1}{\sum }}\cos\theta_{ij}$$

A high value of $DC_{i}$ indicates that individual *i* has a high chance of being socially connected with other individuals, and thus can potentially be successful in influencing others’ health behaviors. This would suggest prioritizing individuals with high values of $DC_{i}$ when recruiting community leaders to work for intervention programs. In addition, the community leaders’ well-being can positively influence others. Thus, community leaders can be identified by evaluating both degree centrality and well-being measure for each node. For example, intervention designers may recruit the most degree-central individuals in the top decile of the well-being distribution in the community.

A similar combination of network and health measures can be leveraged to foster cohesion and social support in a community. Social support has long been known as a major determinant of health [20] while social isolation and perceived loneliness have a demonstrated deleterious impact on health [1]. Reducing perceived isolation and loneliness has been a major aim of health interventions in the past several years [21]. Thus, identifying who can help whom and exchange support in a community could be a primary goal for an intervention. Given a community network, this goal can be achieved by deriving possible contact dyads between people with poor and good well-being scores. Such contact dyads should satisfy two criteria: (1) a high likelihood of contact between two nodes; and (2) a large difference between their well-being measures. Accordingly, we define a “support score” between a pair of nodes *i* and *j* as:

(3) $$\;SS_{ij}=\overset{n}{\underset{j=1}{\sum }}\cos\theta_{ij}\times \left|HS_{i}-HS_{j}\right|$$

where *HS_i_* and *HS_j_* denote any health or well-being measure on nodes *i* and *j*, which can be obtained through health surveys, hospital records, and third-party data sources (e.g., health insurance databases)*.* In our subsequent case study (Section 3), we illustrated the collection of health measures *HS* regarding physical well-being, mental well-being, and social isolation of participants. The resulting support score $SS_{ij}$ can be used to identify the best support provider for a specific person with a poor health measure: given person *i* with poor health, the best support provider *j* for this person is the node that maximizes $SS_{ij}$.

### 2.2. Integrating Social Weights into Spatial Statistics

**Figure 1:**
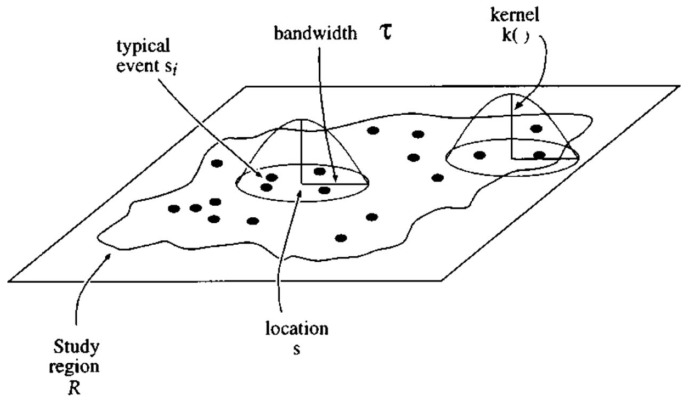
Diagram of how the quadratic kernel density estimation method works, adapted from [23].

Kernel density estimation (KDE) is a widely used spatial statistic to estimate the intensity of a point-based process, e.g., the number of observed homicides per unit area around a given location [22]. Typically, the kernel density estimation is carried out on a grid of locations covering the study area. A kernel-function defined window is centered on each location, and estimates the point intensity around the location (Figure 1). After the kernel visits all locations on the grid, a surface is generated to describe the variation of point intensity (e.g., homicides) over the study area. 

The first step of KDE is to center a kernel at a location *s* on a grid; each observed point event *p* (=1, 2, 3, … *n*) is then assigned a weight according to the kernel function *k(d_sp_)*, which is a function of Euclidean distance *d_sp_* from location *s* to each point *p*. There are many choices for the kernel function *k(d_sp_)*; a common one is the quartic kernel where τ is the bandwidth of the kernel [24]. All points within a distance τ to the location s receive a non-zero weight in the estimation; points beyond this distance are not considered (zero weights). In addition, the value of interest at the point *p, M_p_* (e.g., the number of homicides at *p*), can also be included as a part of the weight. The density estimate at location *s* is the sum of the individual weight made from each observed point *p* (Equation (4)).

(4) $$\hat{\lambda_{s}}=\overset{n}{\underset{p=1}{\sum }}k\left(d_{sp}\right)\times M_{p}$$

where: $k\left(d_{sp}\right)=\frac{3}{\pi \tau^{2}}{\left(1-\frac{d_{sp}^{2}}{\tau^{2}}\right)}^{2};\;d_{sp}<\tau$ [24].

In the context of community health research, we view the point event *p* as a visit by an individual *i* to point *p*. The value measured at the point event *p* (*M_p_* in Equation (4)) is now calculated as the product of the probability of individual *i* visiting point *p* (*V_ip_* from the location vector) and the health measure of that individual (*HS_i_*):

$$M_{p}=\overset{n}{\underset{i=1}{\sum }}HS_{i}\times V_{ip}$$

Thus, the kernel density estimation (Equation (4)) can be modified as:

(5) $$\hat{\lambda_{s}}=\overset{n}{\underset{i=1}{\sum }}\overset{L}{\underset{p=1}{\sum }}k\left(d_{sp}\right)\times HS_{i}\times V_{ip}$$

This KDE analysis integrates individuals’ social/health characteristics with their spatial distribution and produces a density map that shows hotspots of visits, for example, where people with problems of social isolation (if *HS_i_* is a measure of social isolation) or mental illness (if *HS_i_* is an inverse measure of mental health) are highly likely to visit and cluster. 

To exemplify our socio-spatial approach, we applied this method to data collected as part of a larger community-based research on rural Latino immigrants in three North Florida counties, with an overall goal of promoting well-being. 

## 3. Case Study of Rural Latino Immigrants in North Florida

According to the 2010 census, Latinos in Florida comprise 14.7% of rural and small town population in the state, surpassing African Americans (12.9%) as the largest minority group in rural and small town areas [25]. In the last 10 years, Florida has received many Mexicans settling in rural areas that lack the significant social and health resources offered in urban areas such as Miami [26,27]. These rural Latinos tend to be young, poor, with low level of education and working primarily in farming or construction [27]. Because they often experience discrimination and feel excluded from local communities, rural Latino immigrants may avoid certain social and spatial environments, which places them at risk for social isolation and lower levels of well-being [28,29,30,31,32]. Thus, in this methodological case study, we integrated social and spatial analyses to help inform the development of an intervention that targets social isolation and well-being in rural Latino immigrants in North Florida. Specifically, we aimed to: (1) identify community members who could be recruited as leaders for a well-being intervention; (2) identify dyads of community members who could provide support to each other to promote well-being; and (3) map the intensity of participants’ visits to non-home locations, particularly for those who felt socially isolated.

### 3.1. Data Collection and Geocoding

**Figure A1:**
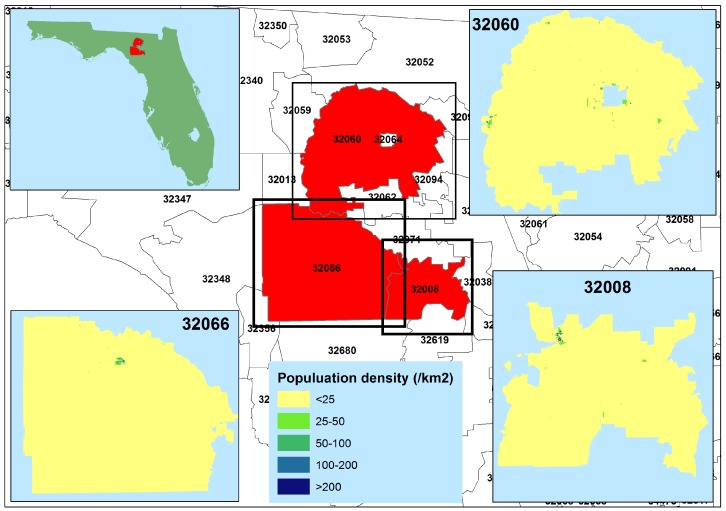
The central map shows the spatial extent of three surveyed communities (ZIP code areas). The upper left map indicates the geographic location of surveyed areas in Florida. Maps in the other three corners depict the density of general population within three communities.

Data used in this case study included three rural communities in North Florida with vast farmland and scattered residences (Appendix A, Figure A1). Participants were rural Latino immigrants at least 25 years old, who had been living in the United States for up to 20 years, resided in one of three rural counties and spoke Spanish. Community engagement principles were used to recruit and to obtain this hard-to-reach population: (1) study team members talked to potential participants during community events in the target rural areas, particularly in schools and churches; (2) the principal investigator received referrals from the community advisory board members; (3) community workers followed up on recruitment attempts; and (4) followed referrals from other study participants. The final sample for this case study was 60 participants (30 couple dyads). The study was approved by the university institutional review board and participants consent were obtained prior the study started. All participants were de-identified during data collection to protect their privacy. The data were stored on an encrypted central server maintained by the university to secure access.

The well-being of participants was assessed using measures of self-rated mental well-being, physical well-being, and perceived social isolation. Self-rated mental well-being of participants was gauged by the mental component score (MCS) of the SF-12v2™ Health Survey, which measures vitality, social functioning, effects of emotional problems on daily activities, and mental health. Self-rated physical well-being was determined by the physical component score (PCS) in the SF-12v2™ survey, which measures the effects of physical problems on daily activities, bodily pain, and general health. *Proprietary* scoring procedures were used to standardize the mental and physical well-being scores between 0 and 100, with a mean of 50 and a Standard Deviation of 10. Higher scores reflected better perception of well-being. 

The perceived social isolation was evaluated through a short version of the PROMIS Health Organization Social Isolation scale, which measures perceptions of being avoided, excluded, detached, disconnected from or unknown by others. The social isolation was also standardized as a score between 0 and 100. A score of 50 is the average for the general population in the United States (Std. Dev. = 10), and higher scores indicate greater feelings of social isolation. 

**Figure A2:**
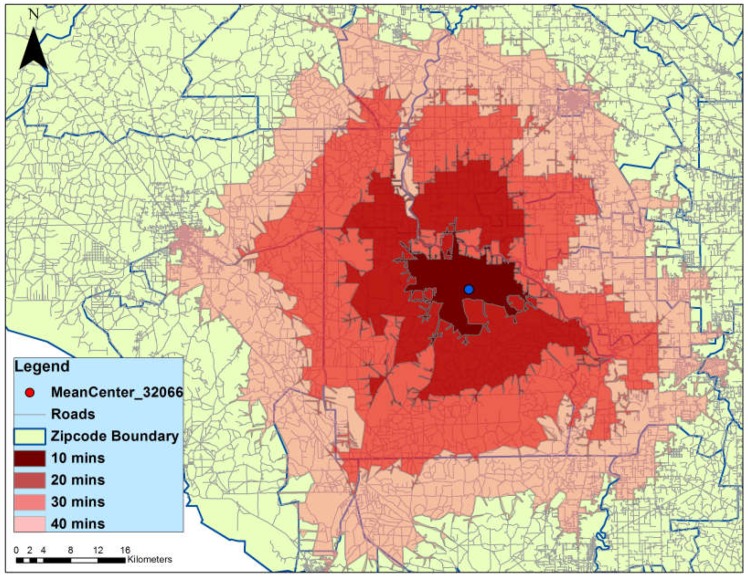
Travel rings of 0–10, 11–20, 21–30, and 31–40 min around the population mean center of a ZIP code area 32066, created based on a real road network.

A questionnaire-based survey was delivered to obtain information regarding participants’ spatial activities. Each participant was asked about routine activities in the past month, including the following questions: *Which ZIP code do you live in? Where have you traveled (e.g., specific places or ZIP codes) in the last month? What are the names of places you have visited in the last month? How long did it take you to get to each place? How often have you been there in the last month?* According to their reports, spatial activities of each participant were geocoded by following three steps: 

(1) *Home location.* To protect identity, the home of each participant was registered to a randomly selected location near to the population weighted mean center of the reported ZIP code (2010 census block populations were used as weights). 

(2) *Other activity places*. To limit respondent burden and to protect respondent privacy, participants were not asked to report specific names or addresses for their activity locations, but only the type of location (e.g., supermarket, church, and park) and their travel time to that location. This information was used to identify potential activity locations, as follows. First, the reported travel time was used to generate a travel ring including all areas that are reachable by car from the participant’s home location within the specified travel time (for example, in 0–10, 11–20, 21–30, or 31–40 min). The road network data of Florida and the ArcGIS network analyst were utilized to create these travel rings (Appendix A, Figure A2). Second, the travel ring was superimposed onto Google Earth to identify potential destinations of the type indicated by the respondent. For example, if “*15 minutes to a church*” was reported for a trip, a travel ring of 11–20 min was generated. All churches shown on the Google Earth and within the 11–20 min travel ring were selected as potential places for this trip. Their latitude and longitude were extracted from Google Earth for subsequent mapping and analysis. Although the workplace is a critical place of daily activities, participants seldom reported this information, perhaps to protect their privacy. Hence, workplace locations were not mapped in this study.

(3) *Visiting probability*. Each potential place was also associated with a daily probability of visit by a participant, computed from the reported frequency of visits by the participant during the last month. Specifically, the daily visit probability was set as 1 if the participant reported visiting the place “every day” (e.g., the home), as 1/7 if the reported frequency was “once a week”, or as 1/30 if “once a month”. If there were multiple potential places for an activity (as identified in Step 2), the daily probability was prorated to each place by dividing it by the number of potential places. The vising probabilities were used to construct the location vector ***V_i_*** for each participant, used in Equation (1). 

### 3.2. Spatially Weighted Social Network

Following the methodology described above, we built a “potential contact” network composed of 60 participants as nodes. For each node, the scores for mental and physical well-being and social isolation were assigned as attributes. Links between participants were created and weighted based on Equation (1), indicating the likelihood of social connections. 

Using this network, we calculated the weighted degree centrality of each node (Equation (2)) to identify potential community leaders, who should have high degree centrality as well as high well-being scores. To find out who can help those with poor well-being, we calculated the “support score” between every pair of nodes (Equation (3)), in terms of mental well-being and social isolation, respectively. We then identified potential supporters to help participants with low mental well-being scores or high social-isolation scores.

### 3.3. Socially Weighted Kernel Density Estimation

Here, we considered each visit to a non-home place by a participant *i* as a point event *p* in Equation (5). We used the social isolation score as the health measure (*HS_i_*) in Equation (5), and thus the kernel density estimation detected hotspots of visits by socially isolated participants. We used ArcGIS Spatial Analyst tool to estimate the kernel density, in which the bandwidth τ was optimized the software to avoid spatial outliers. A greater kernel density around a location indicates more socially isolated people visiting this location. Therefore, outreach and intervention activities at locations with higher density would be more likely to reach disadvantaged pockets of the community.

## 4. Results

### 4.1. Demographics 

**Figure 2:**
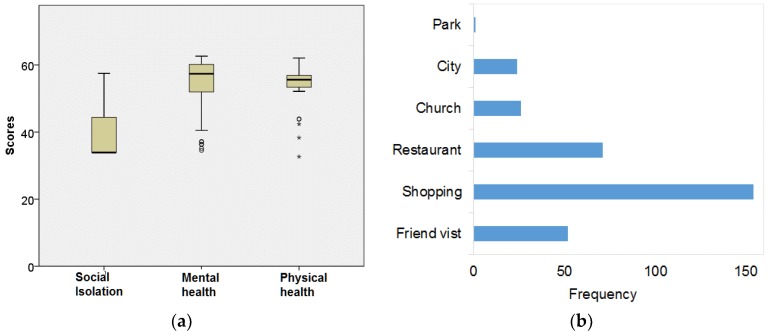
Statistical summary of surveyed data: (**a**) boxplot for three health scores of participants; and (**b**) reported frequency of visiting different types of locations.

Participants were 26–45 years old, primarily from Mexico, who had been living in the United States for more than ten years and had 3–4 children living with them. Women had slightly more education than men, yet the employment rate was higher for men. More information about participants’ demographics can be found in Appendix A, Table 1. The social isolation mean score (39.7) was lower than the national average (50), with a few participants (*n* = 13) scoring above the national average. All scores fell within one standard deviation. The averages of mental and physical well-being scores were 54.2 and 54.4, respectively, both higher than the national average (50), indicating an overall better perception of well-being. There were a few participants with relatively low scores, shown as outliers (Figure 2a). For spatial activities (Figure 2b), “*shopping”* was the most frequently reported activity by participants, followed by “*eating out*”, “*visiting friends*” and “*going to church*”. On average, women visited more places (6.4) in one month than the men visited in the same period (5.2). 

### 4.2. Spatially Weighted Social Network Analysis

**Figure 3:**
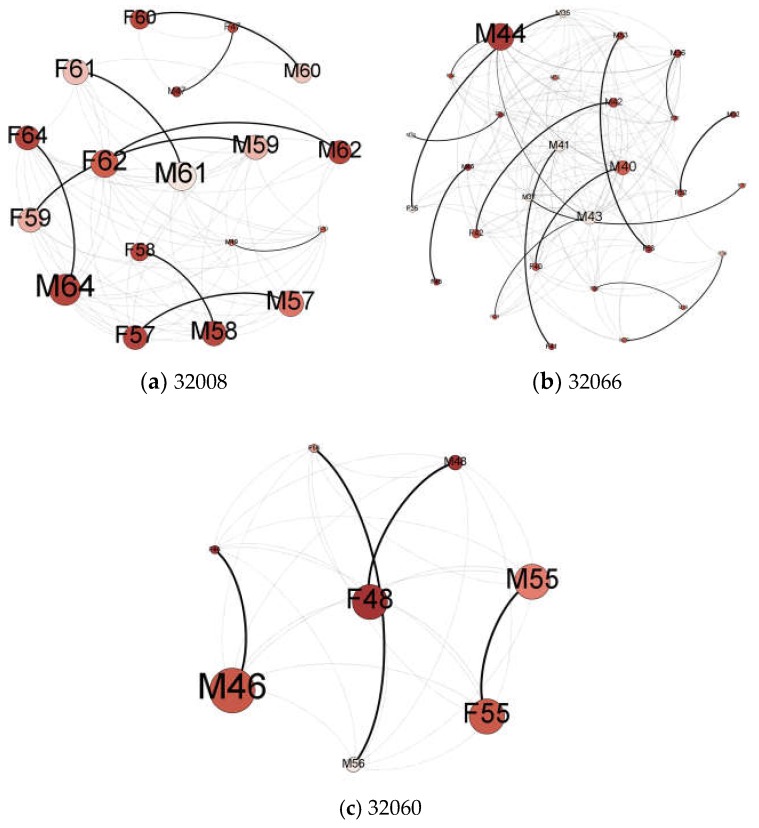
The inferred social network among Latino participants in each of the three communities (ZIP codes). The link width was scaled by the likelihood of social connection. The label “F” stands for “Father” and “M” for “Mother”. The size of nodes indicates their weighted degree centrality, and the tone of color shows their mental well-being scores (the darker the color, the better the mental well-being is). Open-source network analysis software, Gephi, was used to create graphs in Figure 3 and Figure 4.

**Figure A3:**
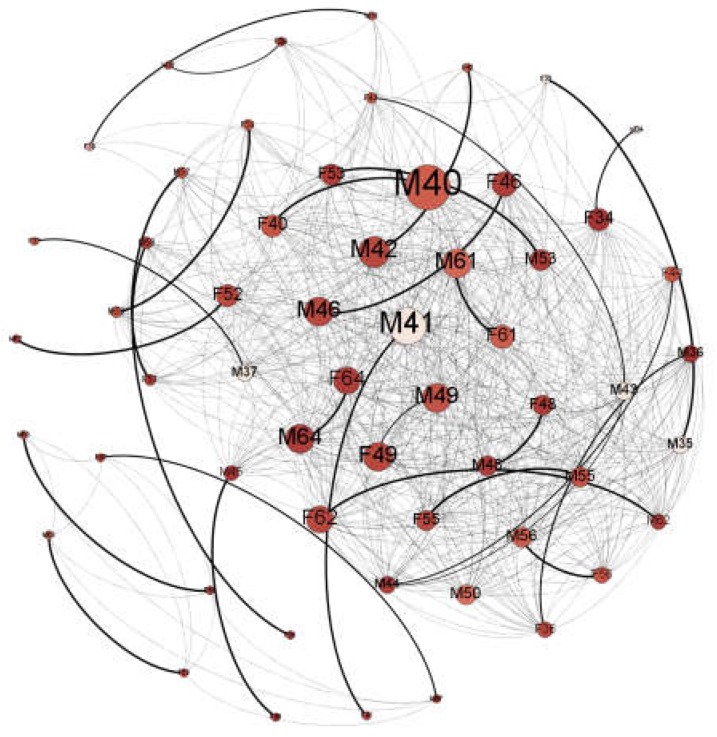
The inferred social network among Latino participants from all three communities combined. The link width was scaled by the likelihood of social connection. The label “F” stands for “Father” and “M” for “Mother”. The size of nodes indicates their weighted degree centrality, and the tone of color shows their mental well-being scores (the darker the color, the greater the mental well-being).

The “potential contact” network derived from this study was composed of 60 nodes (30 couples) and had 1394 links among them. A full structure of this network is visualized in Appendix A, Figure A3. For clearer visualization and analysis, we subset this large network into three sub-networks by ZIP code (community) (Figure 3), which helps identify potential community leaders with high degree centrality with good well-being scores. 

Figure 3 identifies the top two candidate leaders for each community who have high degree centrality and good well-being scores (Table 1).

**Figure 4:**
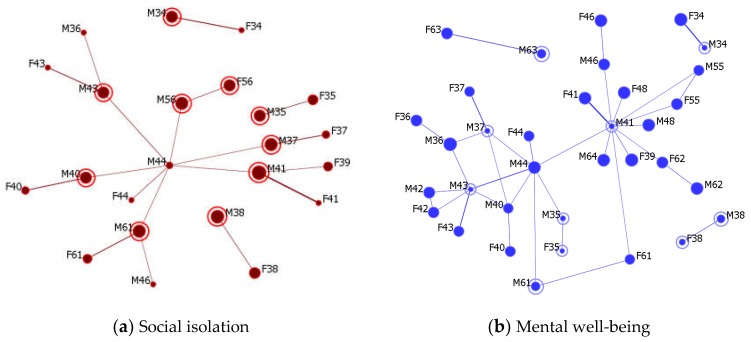
The potential dyads to identify supporters. Node size indicates the social isolation and mental well-being scores, while the links’ width is proportional to the support score. For graph clarity, the links are only shown if their weights (the support scores) are sufficiently large (above 1.0).

Figure 4 depicts possible contact dyads between participants with poor and good well-being scores. Here, “poor” well-being refers to the social isolation score being above 50 or the mental well-being score being under 50. Those nodes with poor well-being were circled in Figure 4 and sized proportionally to their scores of social isolation (Figure 4a) and mental well-being (Figure 4b), indicating those who need more support from a potential intervention. Nodes *i* and *j* are connected by a link whose weight indicates the support score $SS_{ij}$ (Equation (3)). To obtain a clearer network structure, the (*i, j*) link is only displayed if its support score is sufficiently large ($SS_{ij}>1$). For each circled node, the optimal provider of support can be determined by comparing the width of links attached to it. 

### 4.3. Socially Weighted Kernel Density Estimation

**Figure 5:**
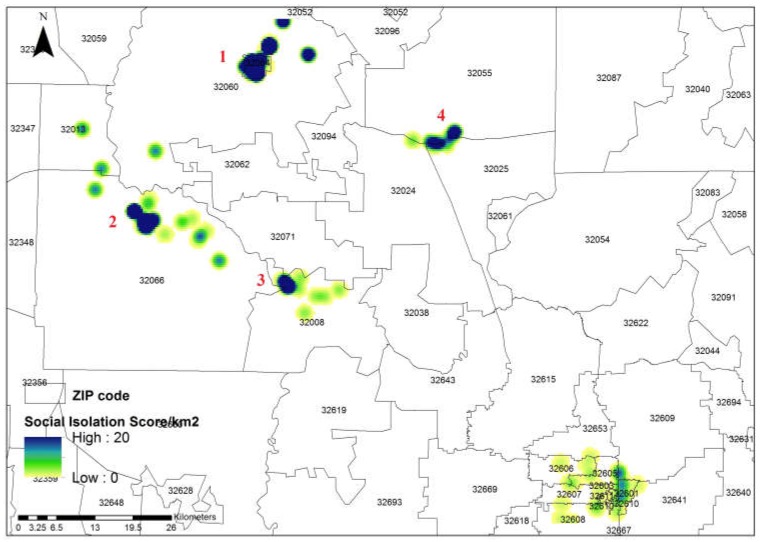
Kernel density map of visiting intensity of socially isolated participants. Dark colored areas indicate locations where participants with high social isolation scores were more likely to visit.

Based on the survey of spatial activities, we identified 110 churches, 101 restaurants, 47 friend visits, and 33 shops (e.g., supermarkets and grocery stores). Generated from the kernel density function, Figure 5 shows four areas with the highest density (dark areas with labels), where visits of participants with high social isolation scores tended to cluster. Areas 1–3 (in the north and northwest) are completely or partially in participants’ home communities (ZIP code areas). Area 4 in the northeast (a city) is about 30–45-minute driving distance to the three studied communities where the survey took place. 

## 5. Discussion

### 5.1. Inferring Social Networks from Spatial Data

In community-based health research, traditional social network analyses rely on self-reports from study participants about their social ties [33], which may face several challenges. First, collection of these data might be excessively costly, time-consuming, or burdensome for respondents, particularly in certain disadvantaged populations (e.g., immigrant farmworkers with limited free time or people with disabilities that prevent them from taking long surveys). Second, self-reports on social interactions might suffer from cognitive biases, privacy issues, errors of perception, and ambiguities [34]. Participants might be reluctant to fully reveal their social connections to protect individuals’ privacy. In certain extreme settings, social network data collection is not feasible or is more strongly affected by respondent accuracy problems. For example, this can be the case if the community of interest is very large and social ties are too many to collect; or if the population under study is stigmatized (e.g., IV drug users) and respondents are reluctant to name other people in the population. To address the challenges, researchers can resort to the method described here and infer networks of potential interactions using several geospatial data sources, such as self-reports on activity locations, geo-tagged photos from Flickr, geo-tagged posts on Facebook, or mobile phone tracking data. Participants, thus, do not have to identify their social ties and expose their privacy. Particularly in a rural community, the population is smaller in size, tend to be less mobile, and has limited places to visit, as compared to its urban counterpart. The co-location of two individuals is more likely to be a real social tie, and thus our inferential approach can produce better prediction of real social network.

In addition to the degree centrality used here (Equation (2)), betweenness centrality and bridging measures can also be calculated in the spatially weighted social network to identify a key lay-community leader. These two measures index the extent to which a node falls in between all other nodes, bridging otherwise unconnected areas of the network [35,36]. Relevant to our case study, participants with high betweenness or bridging values are people who visited a diverse set of locations, which are normally visited by very different groups of people. In other words, high-betweenness individuals have a high likelihood of co-location and potential interaction with different and distant segments of the community, which are otherwise separate or spatially segregated from each other. Thus, in addition to community members with high degree centrality, individuals in bridging positions can also be very effective intervention champions, particularly in more fragmented communities with disconnected subgroups.

### 5.2. Who to Intervene with: Identifying Opinion Leaders and Social Supporters

As shown in Table 1, Respondent M44 in ZIP code 32066, F55 in 32060, and M64 in 32008 all have high mental and physical well-being scores and low social isolation scores. These participants, with their wide connections to other people and positive influence, could assist researchers in the next stage of this study to deliver an intervention and promote the well-being of other community members. 

Although spouses could often be the first candidate for social support, the network graphs in Figure 4 also suggest potential supporters outside individuals’ nuclear families. For example, in Figure 4a, participant M44, the potential community leader of ZIP code 32066 (Table 1), may be an effective support provider for M40, M41, M56, and M37, who all reported high feelings of social isolation. Likewise, M46, as a potential leader in ZIP code 32060, could support M61. The supporters do not necessarily have to be potential community leaders listed in Table 1. For instance, M36 could be a good supporter for M43, and F39 could be a good supporter for M41. Similarly, those who perceived poor mental well-being can find their helpers from Figure 4b.

### 5.3. Where to Intervene: Prioritizing Locations for Outreach and Intervention Efforts 

The kernel density estimation has been widely used to study people’s spatial behavior, but often with no consideration for people’s social or health attributes [16]. In these applications, the identified hotspots only show where people are more likely to visit and cluster in general, but do not differentiate subgroups of interest, such as those who are socially isolated, at risk of mental illness, or with poor well-being. Interventions deployed in these hotspots may be effective at reaching people in general, but may be less likely to reach the subgroups of interest, and thus are less cost-effective. In contrast, by incorporating social or health-related weights into kernel density estimation, hotspots of interest can be more accurately identified, where specific, higher-priority subgroups of people tend to cluster. In our application, the resulting kernel density map (Figure 5) shows that the activities of most socially isolated participants were concentrated in the vicinity of their home locations, but many of them also traveled long distances to medium-size cities (two medium high-density areas in the south and southeast). In those hotspots, we identified three places where socially isolated participants were likely to gather, namely Store A (in ZIP code 32008), Store B (32060), and Store C (32066). These are key places where community interventions can be deployed to more efficiently reach higher numbers of individuals in priority populations.

Although we focused on social isolation, similar analyses can be performed for mental and physical well-being, which are not illustrated here. Differently, the health score *HS_i_* in Equation (5) should be an inverse measure of health or well-being, such that participants with lower well-being have higher *HS_i_* scores and higher weights (resulting in higher kernel density). For example, if *MCS_i_* is the mental component score of the SF-12v2™ Health Survey, then we could define *HS_i_* = 100 – *MCS_i_* for KDE analysis.

### 5.4. Limitations

Three limitations of this study should be noted. First, part of the analysis was based on a social network inferred from co-locations of participants’ activities. This should be interpreted as a network of *potential* face-to-face encounters and interactions among participants, due to the spatial proximity of their daily locations; however, this network does not necessarily reflect *actual* face-to-face contacts among individuals. Participants who appear as connected in the network do not necessarily know or trust each other. The actual network of acquaintances, friendships or communications among participants could be different. This limitation should be kept in mind when interpreting the meaning of centrality measures, community leaders, or support scores derived from the network. Nevertheless, the spatial inferential approach has been increasingly adopted by network-related health studies, and is considered reliable and valuable when actual social network surveys are not feasible [19,33,37]. 

Second, the identification and geocoding of participants’ activity locations was at times problematic. Many participants reported vague names for places they had visited, and were reluctant to provide their workplace information, which made their complete activity space difficult to map. To address this problem, our method assigned all possible locations of the specified type and within the specified travel ring to each participant’s activity space. Although this ensures that individual privacy is protected, the incomplete information can introduce uncertainty in the structure and analysis of the resulting spatially-weighted network and kernel density estimation. This is, however, a data problem that can be addressed using GPS equipped devices or Google Map during the survey, and does not affect the validity of our proposed approach.

Third, the sample size is relatively small in the socio-spatial analysis (30 women and men living together) and not random, which is typical of hidden or hard-to-reach populations such as immigrants in rural areas [38]. Our approach, however, can handle a larger sample and produce results that are more reliable. While all these limitations suggest that a more sophisticated and larger-scale survey would be beneficial in community health applications, they do not diminish the value of the integrated socio-spatial analysis approach. Such an approach seems a promising methodological tool for studying health disparities and designing health interventions in underrepresented and isolated communities.

## 6. Conclusions

Geographic space and social space are closely intertwined, and research should attempt to analyze them together to identify the “who” and “where” of health intervention endeavors. Previous studies have mostly focused on one of the two spaces, overlooking their interaction and the additional insights that can be gained from an integration. This suggests that there is much room for improving intervention programs by leveraging results from joint socio-spatial analyses. We offered one proposal for a socio-spatial analytic approach that integrates spatial co-location into social network analysis, and social/health measures into spatial kernel density estimation. Other ways to combine analyses of social and spatial data are certainly possible, and future research in community, public and rural health would greatly benefit from further investigating such integration. We believe that this line of work would enrich the methodological body of community health research, and bring real team-based science contributions, particularly when working with hard-to-reach rural populations, such as: (1) optimizing recruitment, retention and eliminating barriers to participation of minority and rural populations in community interventions; (2) facilitating community partnerships with individuals and organizations; and (3) assisting in negotiating intervention sustainment, and minimizing socio-spatial inequities for community-based interventions engagement.

## Figures and Tables

**Table 1:** Top 2 potential community leaders by ZIP code area and their social-health measures.

Node ID ^a^	ZIP Code Area	Degree Centrality	Mental Well-Being Score	Physical Well-Being Score	Social Isolation Score
M44	32066	1.69	60.16	55.85	39.1
M42	32066	1.25	51.96	57.17	43.1
F55	32060	1.23	57.38	57.12	33.9
M46	32060	1.24	57.53	54.64	33.9
M64	32008	1.29	60.32	53.37	33.9
F62	32008	1.18	57.53	54.64	39.1

**^a^** M stands for mother, while F stands for father.

## Appendix A

**Table A1:** Demographic summary of participants ^a^.

	Women (*n* = 30)	Men (*n* = 30)
**Country of Origin** El Salvador Mexico Guatemala	6.7% 90.0% 3.3%	6.7% 93.3% 0%
**Language** (Spanish)	100%	100%
**Years in US** 10 or less More than 10	13.3% 86.7%	16.6% 83.4%
**Age** 26–35 36–45 46–65	33.3% 53.4% 13.3%	33.3% 50% 16.7%
**Number of children *** 1–2 3–4 5–6	24.1% 65.5% 10.4%	26.7% 66.7% 6.6%
**Children in country of origin **** No Yes	92.9% 7.1%	89.7% 10.3%
**Employed** No Yes	33.3% 66.7%	3.3% 96.7%
**Years of education** 0 1–4 5–8 9–14	0% 20% 36.7% 43.3%	6.7% 13.4% 46.6% 33.3%
**Family/friends nearby** No Yes	13.3% 86.7%	6.7% 93.3%
**Mean number of places visited in last month *****	6.4	5.2
**Mean number of cities visited in last month ***	2.9	2.5

Note: ^a^ This table is adopted from a previous publication [39]. * 1 missing, valid percentages used, ** 2 missing, valid percentages used, *** >2 missing.
